# Supplementary material for: Ramping dynamics and theta oscillations reflect dissociable signatures during rule-guided human behavior
Source: Nat Commun. 2024 Jan 20;15:637. doi: 10.1038/s41467-023-44571-7 (PMC10799948; doi:10.1038/s41467-023-44571-7)
Supplement: Supplementary file 1 — Supplementary Information [file 41467_2023_44571_MOESM1_ESM.pdf]

Supplementary Information for:

Ramping dynamics and theta oscillations reflect dissociable signatures during rule-guided human behavior.

Jan Weber[1,2], Anne-Kristin Solbakk[3,4,5,6], Alejandro O. Blenkmann[3,4], Anais Llorens[3,4,7], Ingrid Funderud[3,4,6], Sabine Leske[3,4,8], Pål Gunnar Larsson[5], Jugoslav Ivanovic[5], Robert T. Knight[7,9], Tor Endestad[3,4,10], Randolph F. Helfrich[1,10,*]

[1] Hertie Institute for Clinical Brain Research, Center for Neurology, University Medical Center Tübingen, Tübingen, Germany.

[2] International Max Planck Research School for the Mechanisms of Mental Function and Dysfunction, University of Tübingen, Tübingen, Germany.

[3] Department of Psychology, University of Oslo, Oslo, Norway

[4] RITMO Centre for Interdisciplinary Studies in Rhythm, Time and Motion, University of Oslo, Oslo, Norway

[5] Department of Neurosurgery, Oslo University Hospital, Oslo, Norway

[6] Department of Neuropsychology, Helgeland Hospital, Mosjøen, Norway

[7] Helen Wills Neuroscience Institute, UC Berkeley, Berkeley, CA, USA

[8] Department of Musicology, University of Oslo, Oslo, Norway

[9] Department of Psychology, UC Berkeley, Berkeley, CA, USA

Supplementary Note

Population dynamics in PFC encode context and actions in serially unfolding subspaces. While it is well established that neural oscillations reflect coordinated population activity[1], the population correlates of ramping dynamics are less understood. Having established that ramping dynamics index context-dependent activity, we assessed how ramping dynamics impact population dynamics. In neural state space, population dynamics can be conceptualized as a trajectory across time through a N-dimensional space (Methods). We computed the multidimensional distance (MDD) between pairwise trajectories. We observed that neural state-space trajectories in PFC were context-dependent and diverged prior to participants' choice of behavioral response (button release; Supplementary Fig. 6a; $t(16) = 46.13$, $p = 0.024$; cluster test). This effect was driven by strong state transitions in trials with high behavioral uncertainty (Supplementary Fig. 6a; $F(2,32) = 96.01$, $p = 0.011$; cluster test). No statistically significant modulation was found for a context-dependent evolution of neural trajectories in motor cortex (Supplementary Fig. 6b; $t(13) = 13.69$, $p = 0.116$). Instead, the neural state transitions did not statistically differ between the different conditions as indicated by similar pattern across all three levels of uncertainty (Supplementary Fig. 6b; $F(2,26) = 23.81$, $p = 0.123$).

We extracted the most dominant, population-wide activity pattern using principal component analysis (PCA) to examine the latent dynamics underlying context-dependent computations (Supplementary Fig. 6c/d/e). We found that the top three principal components (PCs) captured 35.76 ± 14.15% of the variance (mean ± SD) in PFC and 50.6 ± 24.88% of the variance in motor cortex (Supplementary Fig. 6f; $t(13) = -2.2$, $p = 0.046$, Cohen's $d = 0.66$; two-tailed t-test). We also observed that the variance explained per PC decreased faster in motor cortex as compared to PFC ($t(13) = -2.01$, $p = 0.065$, Cohen's $d = 0.67$; two-tailed t-test), suggesting that neural dynamics are higher dimensional in the human PFC than motor cortex. In line with this finding, population activity in PFC (PC1) revealed context-dependent dynamics (Supplementary Fig. 6c; $F(2,32) = 1.18 \times 10^3$, $p = 0.008$; cluster test) with larger activation states in trials with high behavioral uncertainty. In comparison, we found that latent dynamics across all context conditions did not differ significantly in motor cortex (Supplementary Fig. 6d; $F(2,26) = 230.85$, $p = 0.079$). This set of findings indicates that population activity in PFC is modulated by predictive context. In contrast, motor cortex population activity might rather reflect context-invariant dynamics supporting a condition-invariant signal[2] (see Discussion). We additionally time-locked the population activity to the HLL to ensure that context-dependent activity in PFC ramped up and was dissociable prior to the HLL and not a consequence of an activation change triggered by the HLL (Supplementary Fig. 7/8). Next, we tested whether predictive context also modulates the inter-areal coupling in the prefrontal-motor network on a population level. We quantified the degree of functional connectivity between population activity in PFC and motor cortex on a single-trial level (Methods). This revealed that the strength of the prefrontal-motor

interaction scaled with behavioral uncertainty (inset Supplementary Fig. 6e; $F(2,26) = 4.69$, $p = 0.018$, $\eta_p^2 = 0.27$; one-way RM-ANOVA) and explained behavior on a single trial basis ($F(1,2410) = 35.76$, $R^2 = 0.014$, $p < 0.001$; linear regression). Collectively, we have shown that the activation state of population dynamics in the human PFC gradually scales as a function of uncertainty.

In order to better visualize and contextualize the univariate results (Figures 1-4) and to identify behaviorally-relevant factors in the state space, we employed a multivariate approach to trace the temporal evolution of different behaviorally-relevant factors along the prefrontal-motor hierarchy. Therefore, we assessed which latent dimension reflects contextual encoding in human PFC, where population dynamics are high-dimensional. To determine the relevant coding dimensions, we employed a Linear Discriminant Analysis (LDA) classifier in PC space, separately for both regions. This approach defined the coding dimensions that maximally discriminated context and reaction times (split into terciles; referred to as action-encoding; Methods). This approach dissociates dynamics that are relevant for contextual processing and subsequent action planning. Note that we were not primarily interested in distinguishing fast vs. slow RTs, but employed the classifier as a tool to trace the temporal evolution of the representation of the overt behavioral response. We time-locked the population response to the timing of movement execution to mitigate the impact of distinct cognitive states at the HLL during different contexts. Thus, neural activity that dissociates reaction times prior to movement execution (and that is not primarily driven by early context-integration signals) likely reflects the internal transition from the planning to the execution of a movement. To separate these two processes, we constrained the coding dimensions of context and action to be orthogonal in neural state space (Methods). We found that human PFC encoded both context and action (Supplementary Fig. 6g; context: $t(16) = 352.99$, $p < 0.001$, Cohen's $d = 0.83$; action: $t(13) = 1445.56$, $p < 0.001$, Cohen's $d = 1.4$; cluster test). Critically, context could be decoded prior to action (Supplementary Fig. 6g; context: 443ms prior to button release; action: 234ms prior to button release), suggesting that context was integrated before translation into an action plan. Importantly, we found that the coding dimensions maximally discriminating context and action planning mapped onto distinct PCs in 11/14 participants ($p = 0.057$; Binomial test), suggesting that these processes are dissociable in the neural state space. In contrast, we were able to reliably discriminate action-timing, but not context, from motor cortex (Supplementary Fig. 6g; context: $t(13) = 39.32$, $p = 0.485$, Cohen's $d = 0.75$; action: $t(9) = 1118.44$, $p < 0.001$, Cohen's $d = 1.39$; cluster test), indicating that the relevant contextual computations might have been completed prior to the output stage. We performed an additional control analysis to preclude that the action-decoding was confounded by capturing different periods of the task (i.e., pre-HLL for fast RTs, post-HLL for slow RTs) when locked to the BR. Therefore, we additionally time-locked the population response to the HLL and repeated the classification analysis. In this case, the linear decoder captures the same periods of the task regardless of the respective RT. This analysis revealed a build-up of action-specific information prior to the HLL

(Supplementary Fig. 9; PFC: $t(9) = 1074.87$, $p < 0.001$, Cohen's $d = 1.69$; motor cortex: $t(6) = 322.79$, $p < 0.001$, Cohen's $d = 1.67$; cluster test), therefore, supporting the notion that the decoder captured action-planning processes. We performed additional control analyses demonstrating that the context-encoding and action-encoding subspaces capture dissociable processes related to contextual computations and the planning of actions (Supplementary Fig. 10; Methods). We also performed the decoding analyses using a high-dimensional neural space on all principal components cumulatively explaining more than 95% of the variance. This replicated our main findings that the human PFC encodes both context and action (Supplementary Fig. 11; context: first cluster, $t(16) = 408.68$, $p = 0.002$, Cohen's $d = 0.74$; second cluster, $t(16) = 151.62$, $p = 0.039$, Cohen's $d = 0.69$; action: $t(16) = 1040.76$, $p < 0.001$, Cohen's $d = 1.08$; cluster test) whereas motor cortex only significantly encodes action (Supplementary Fig. 11; context: first cluster, $t(13) = 39.46$, $p = 0.427$, Cohen's $d = 0.66$; action: $t(13) = 828.57$, $p < 0.001$, Cohen's $d = 1.11$; cluster test). Having established that context and action can be decoded from the prefrontal-motor network, we next quantified their temporal dynamics by computing the peak-decoding latency. Peak-decoding accuracy revealed a distinct temporal pattern with initial information about contextual information in PFC (Supplementary Fig. 12; $p = 0.024$; Kruskal-Wallis; -0.09 ± 0.19s. with respect to BR; mean ± SD), followed by action-related processes in PFC (0.05 ± 0.15s.) and motor cortex (0.11 ± 0.12s.), suggesting that contextual computations in PFC are possibly completed prior to action-relevant computations. Although decoding accuracy for action reached its maximum right after the movement onset, we observed a build-up of action-related information over time, suggesting that the decoder likely captures the direct transition between action-planning and action-execution.

This raises the question, whether and how the representation of the action plan in PFC is passed on to motor cortex for final action execution? To address this question, we first computed the direction of propagation of neural activity between action encoding subspaces in PFC and motor cortex using cross-correlation analysis (Methods). This revealed a significant time lag of activity between the action encoding subspaces ($p = 0.019$; -18.16 ± 16.34ms; mean ± SD; Wilcoxon rank-sum test; two-tailed). Activity within the PFC action subspace significantly preceded activity within the action subspace in motor cortex, demonstrating that action-relevant processes in PFC temporally precede those in motor cortex.

To infer whether action-related information generalizes from PFC to motor cortex prior, we conducted a cross-regional pattern analysis (Methods). We trained a linear classifier on the activity of the PFC action subspace to discriminate reaction times and subsequently tested if the classifier generalized to the action subspace in motor cortex. Hence, we tested if a discriminative pattern that was present in the action-specific subspace in PFC was equally present in the action-specific subspace in motor cortex. We employed a time-generalization decoding approach, since we predicted that a cross-regional generalization should occur at a lag if this truly constitutes a transfer of action-related information. In this

scenario, above-chance classification along the diagonal indicates that the information between PFC and motor cortex is temporally specific without delay, whereas off-diagonal classification indicates the presence of a temporal lag. A linear decodes trained on the PFC action subspace generalized to the action subspace in motor cortex with a temporal delay (Supplementary Fig. 6h; $t(8)$ = 70605, p = 0.002, Cohen's d = 0.64), providing further evidence that action-specific information generalized from PFC to motor cortex.

In sum, this set of findings supports the view that the human PFC integrates contextual information at early stages in a low-dimensional subspace and devises them into an appropriate action plan that is subsequently executed in motor cortex.

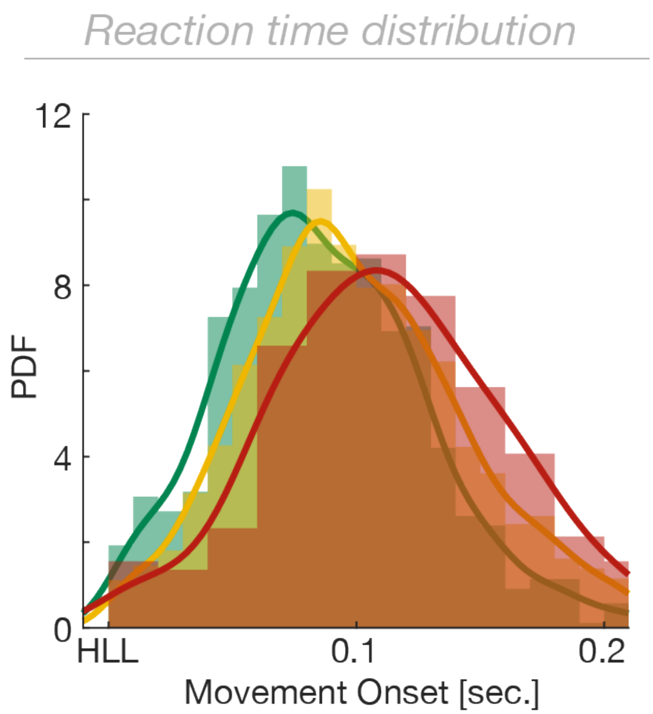

Supplementary Figure 1. Reaction time distribution. The distribution depicts the movement onset with respect to the lower limit (HLL) for the different contexts (green = 0%; orange = 25%; red = 75%). Source data are provided as Source Data file.

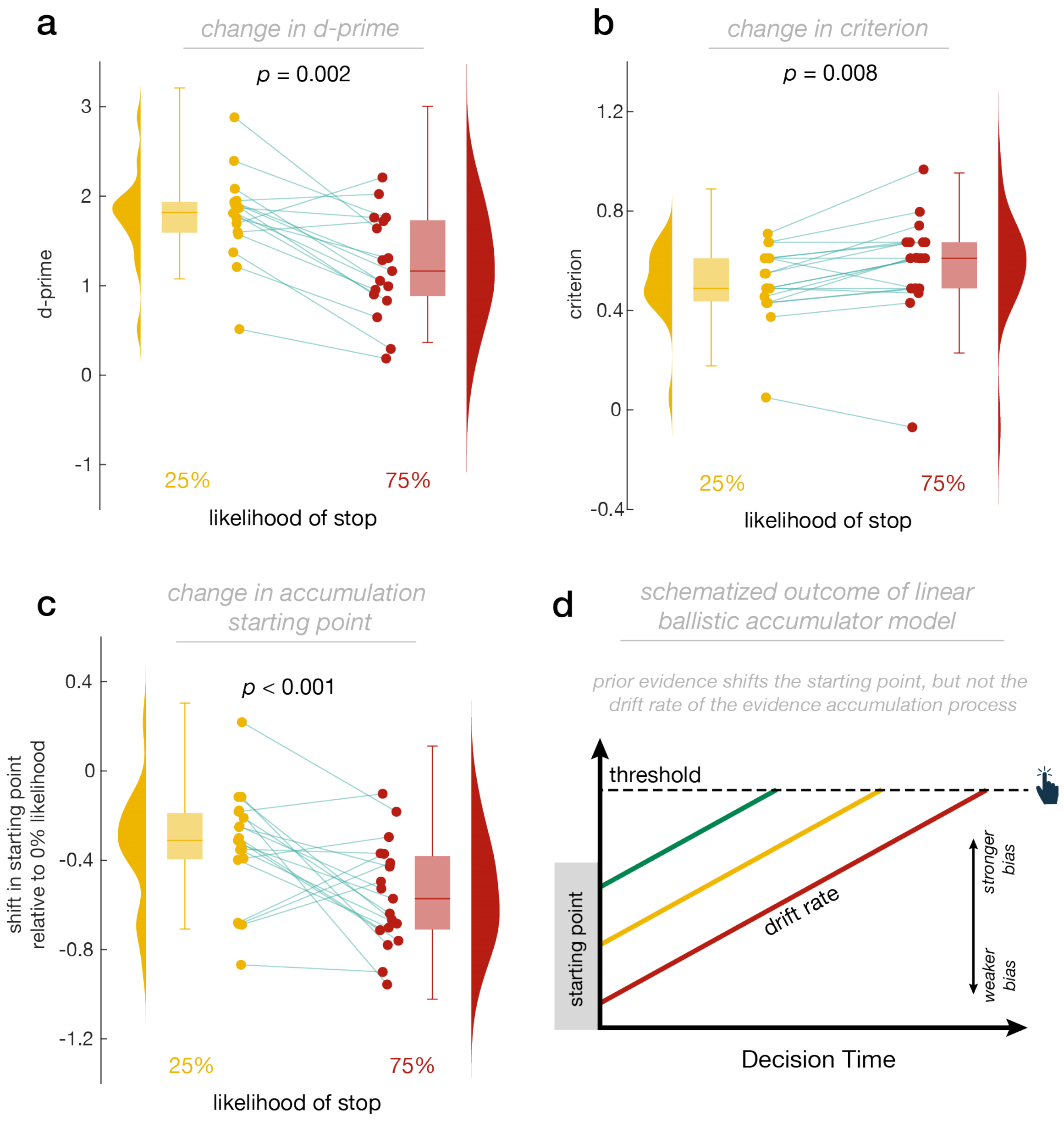

Supplementary Figure 2. Prior evidence modulates the decision strategy. a The predictive cue significantly shifted d-prime, reflecting an increased separation between the internal signal and noise distribution (p = 0.002, n = 17; Wilcoxon signed-rank test; two-tailed). Grey lines display individual participants, density-plots display the data distribution and boxplots show the median (horizontal line), the first/third quartile (upper/lower edge of box) and the minima/maxima (vertical lines). **b** Participant's response bias (criterion) significantly increased during trials with high uncertainty, suggesting a more conservative response strategy (p = 0.008, n = 19; Wilcoxon signed-rank test; two-tailed). **c** The starting point was significantly reduced when participants received less prior evidence, suggesting that more evidence had to be acquired to make a response (p < 0.001, n = 19; Friedman test). **d** Schematized outcome of the linear ballistic accumulator model.

Prior evidence shifted the starting point, but not the drift rate of the evidence accumulation process. Source data are provided as Source Data file.

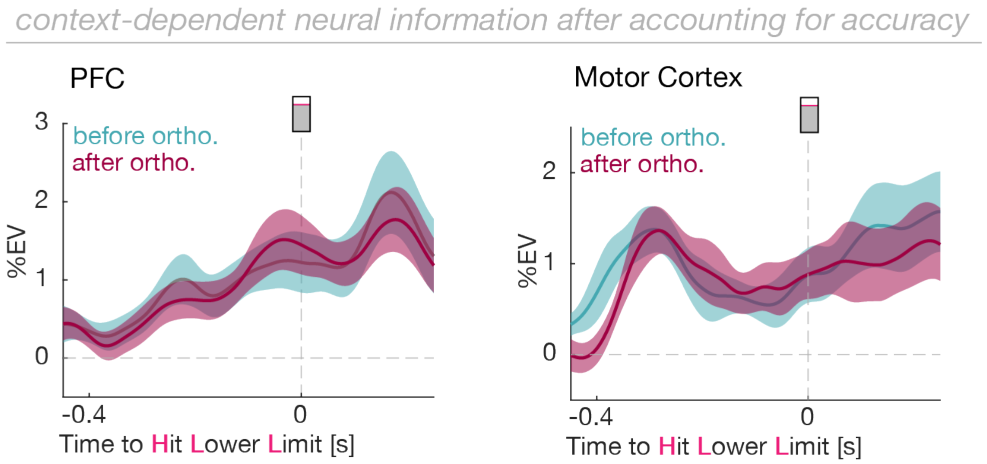

Supplementary Figure 3. Context-dependent neural information cannot be explained by accuracy. An unbalanced ANOVA, that implicitly orthogonalized the factors *context* and *accuracy*, was computed. Thus, variance explained by context cannot be explained by accuracy, or vice versa. Context-dependent neural information, however, is neither significantly altered in PFC (left; n = 16) nor motor cortex (right; n = 11) after orthogonalization (cluster test). Hence, the context-dependent neural information cannot be explained by accuracy. Lines and shaded regions show the mean and SEM. Source data are provided as Source Data file.

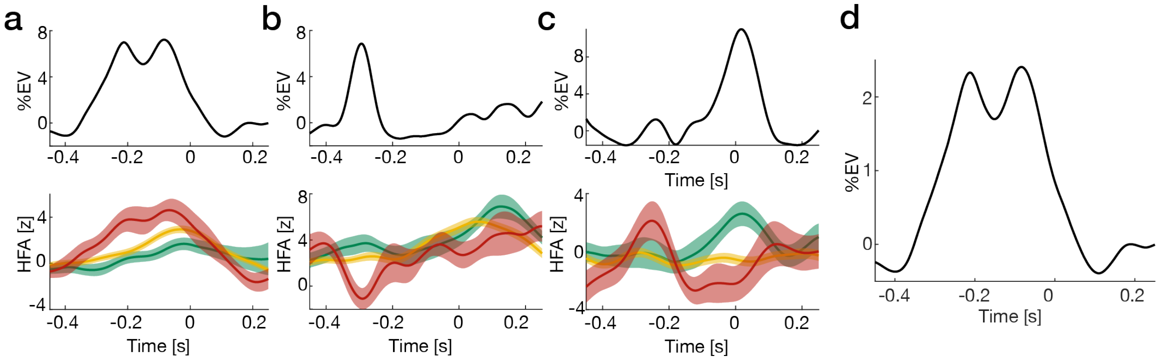

Supplementary Figure 4. Single electrode examples of percent explained variance and underlying high-frequency activity traces. a-c Single electrode examples from motor cortex showing significant context-dependent information (percent explained variance; %EV; top row). Note however, that the direction of the effect differs across electrodes (bottom row), e.g. while the explained variance in panel **(a)** is driven by a gradual change in HFA from low to high states of uncertainty (green = 0% likelihood; orange = 25% likelihood; red = 75% likelihood), this is not visible in single electrode in panel **(b)** and **(c)**. Yet, these directional differences on the effect cannot be dissociated solely based on the percent explained variance (PEV) as this metric is blind with respect to the direction of the effect. **d** *Top:* PEV averaged across examples shown in **(a-c)**. Note that the effects from **(b)** and **(c)** are largely cancelled out on the grand average because the effect occurred at distinct timepoints. Yet, on average, variability in neural activity still explains up to 4.48% variance of the task-feature, although individual electrodes strongly differed in terms their selectivity. *Bottom:* shows the averaged neural response across all three example electrodes per condition. Because of the variability between the electrodes, averaging them largely cancels out the effects.

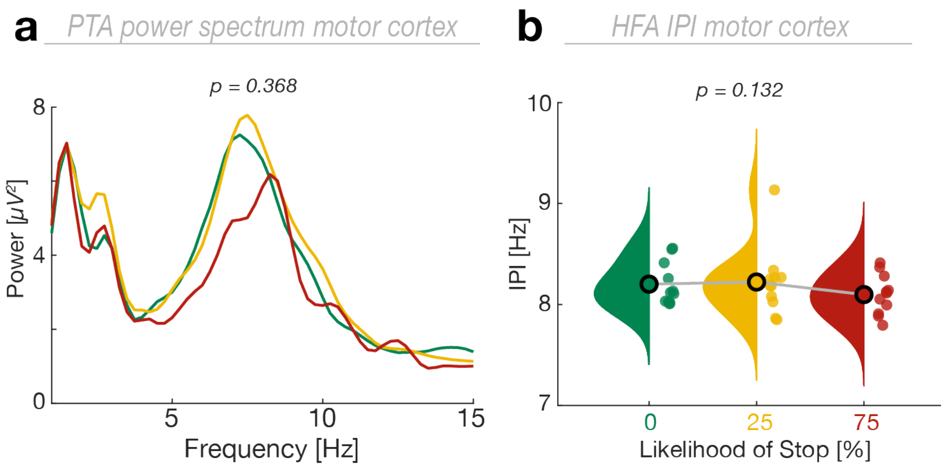

Supplementary Figure 5. Theta oscillations in motor cortex are not modulated by predictive context. a Grand average 1/f-corrected power spectrum computed on the peak-triggered-average (PTA) time-series using IRASA. The HFA was strongly nested in theta oscillations across all three predictive context conditions as can be seen by the pronounced theta peaks in the power spectrum ($F(2,20) = 7.61$, $p = 0.368$, n = 11; cluster test). **b** The HFA inter-peak-interval in motor cortex was not significantly modulated by predictive context ($F(2,20) = 2.24$, $p = 0.132$, n = 11; one-way RM-ANOVA). Source data are provided as Source Data file.

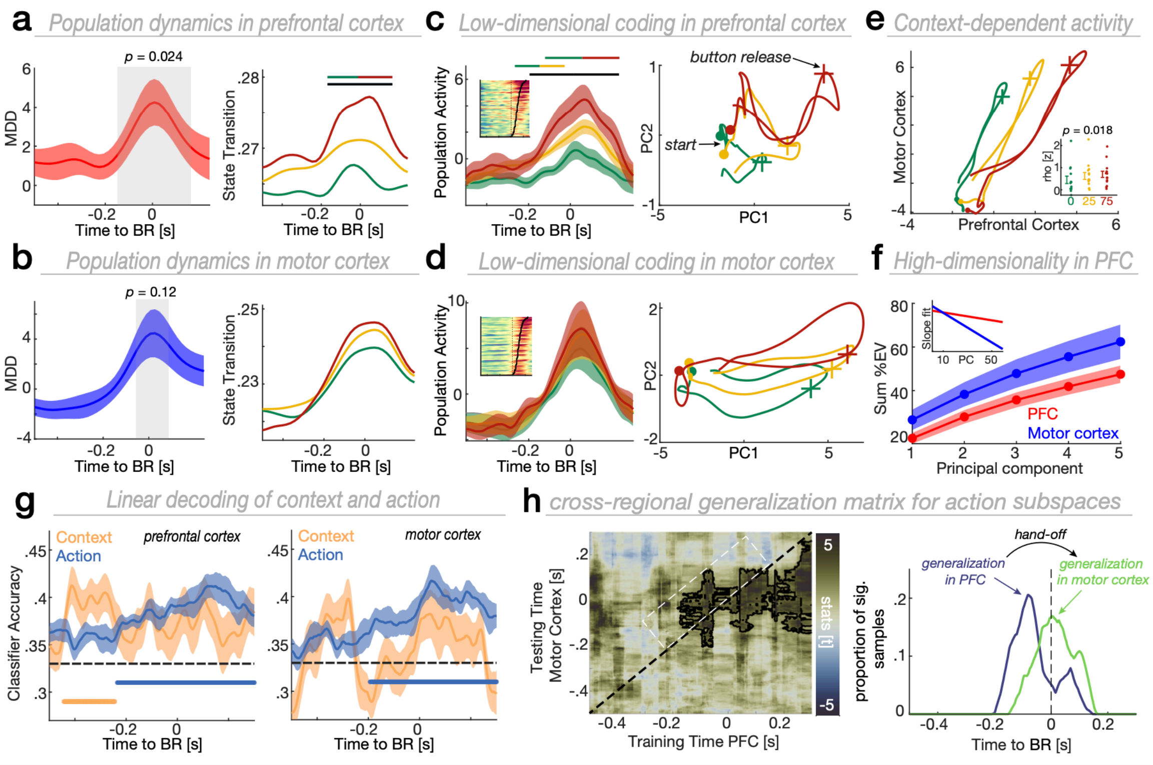

Supplementary Figure 6. Representation of context and action in distinct coding dimensions. a Left: Multidimensional distance (MDD; mean ± SEM) between the different contexts in the in PFC, highlighting a significant (grey shaded) divergence before the response ($t(16) = 46.13$, $p = 0.024$, n = 17; cluster test; two-tailed). Right: Within-condition state transitions (n = 17). Horizontal lines indicate significant context dependency. The multicolored lines indicate pairwise comparisons (cluster test; two-tailed). **b** Left: MDD in motor cortex ($t(13) = 13.69$, $p = 0.116$, n = 14). Right: Within-condition state transitions (n = 14). Same conventions as in **(a)**. **c** Left: Dissociation of context in the first principal component (PC1; n = 17; cluster test). Same conventions as in **(a)**. The inset depicts stacked single trials sorted by RT. Right: Low-dimensional neural trajectories in PFC (first two PCs; filled dots indicate the start; the crosses indicate the BR). **d** Left: PC1 did not dissociate context conditions in motor cortex. Same convention as in **(c)**. **e** Projection of PC1 in PFC and motor cortex into a common 2D space. Same conventions as in panel **(c)**. Inset: z-transformed power correlations between prefrontal-motor activity ($F(2,26) = 4.69$, $p = 0.018$, n = 14; one-way RM-ANOVA). Dots represent data from single participants. Error bars indicate SEM. **f** Cumulative percent variance (%EV; mean ± SEM) explained by the top five PCs in PFC (red) and motor cortex (blue). The %EV decayed more rapidly in motor cortex (inset). **g** Left: Decoding accuracy (mean ± SEM) for context and action within PFC. Horizontal lines indicate significant clusters (same color conventions; cluster test; two-tailed). Right: Decoding accuracy for context and action within motor cortex. **h** Left: cross-temporal classification between

action subspaces in PFC and motor cortex (black outline indicates significance; cluster test; two-tailed). Significant cross-temporal classification is dominant along the off-diagonal. Prior to the BR, cross-temporal generalization was mainly observed along the upper triangular, indicating a temporal delay from PFC to motor cortex. Right: Average cross-temporal classification in the upper triangular reveals a temporal lag of 105ms between PFC and motor cortex. Source data are provided as Source Data file.

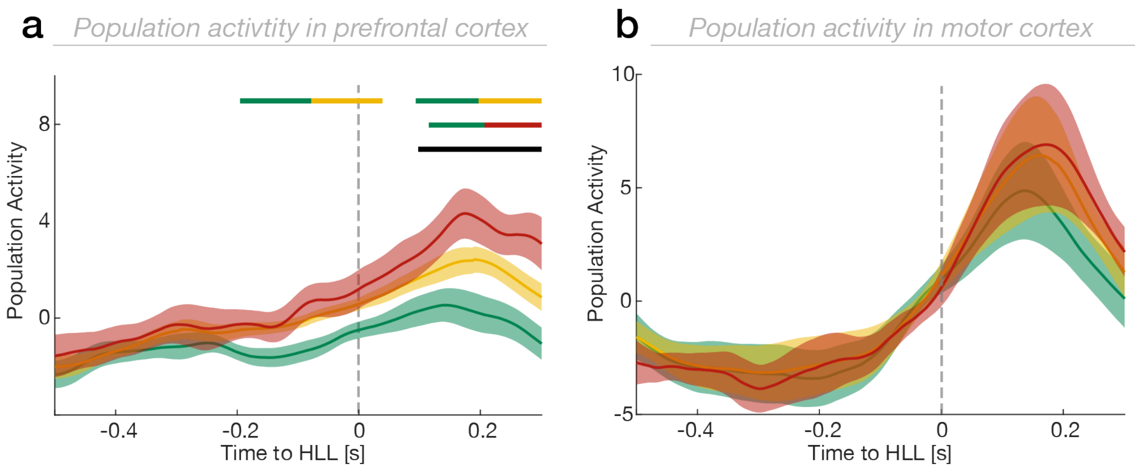

Supplementary Figure 7. Context-dependent population dynamics in PFC emerge prior to the HLL. a Population activity in PFC as indexed by the first principal component reveals context-specific activation patterns prior to the onset of the HLL ($F(2,32) = 637.4$, $p = 0.023$, n = 17; cluster test). The single-colored horizontal lines show the temporal extent of a significant context-dependent dissociation. Two-colored horizontal lines indicate the temporal extent of significant clusters obtained from pairwise comparisons (cluster test; two-tailed). Shading depicts the SEM across participants. **b** Same as **(a)**, but for motor cortex. Note the similar, context-independent, temporal activation profile across all conditions in motor cortex ($F(2,26) = 251.7$, $p = 0.073$, n = 14; cluster test). Same conventions as in **(a)**. Source data are provided as Source Data file.

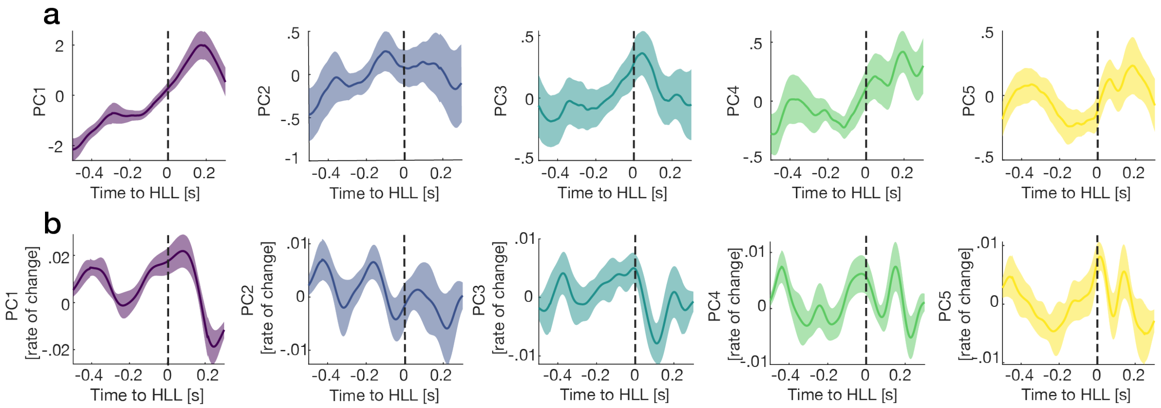

Supplementary Figure 8. Population dynamics quantified by the first 5 principal components do not show a change in activity triggered by the lower limit. a Neural dynamics in PFC as indexed by the principal component time-series (1 to 5) do not show a time-locked change upon the lower limit. **b** We computed the temporal derivative of the traces in **(a).** This revealed that there is no strong rate of change in neural activity upon the lower limit. Source data are provided as Source Data file.

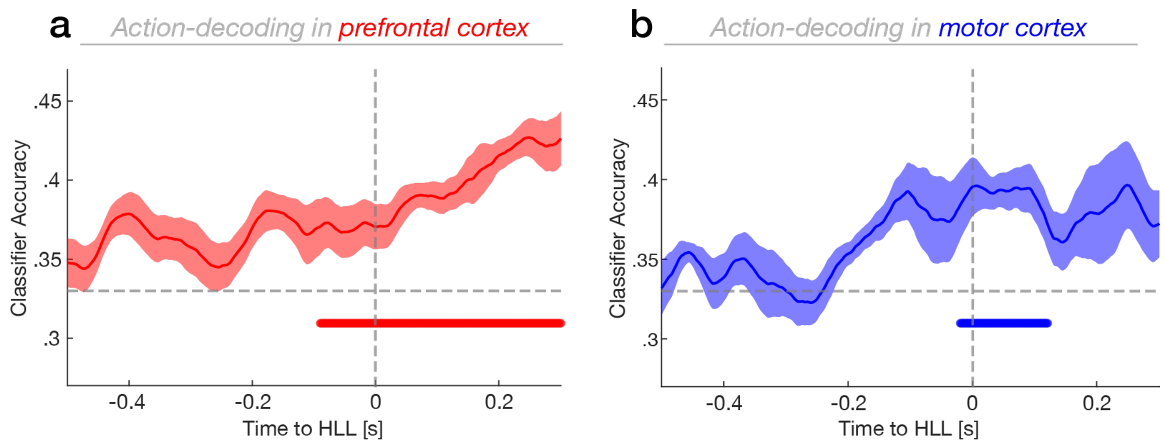

Supplementary Figure 9. Action-specific information emerges prior to the HLL. a, Grand average decoding accuracy (mean ± SEM) for action within PFC when locked to the HLL. Horizontal lines indicate the extent of significant temporal clusters (n = 10; cluster test; two-tailed). **b** same as **(a)** but for motor cortex (n = 7). Same conventions as in **(a)**. Source data are provided as Source Data file.

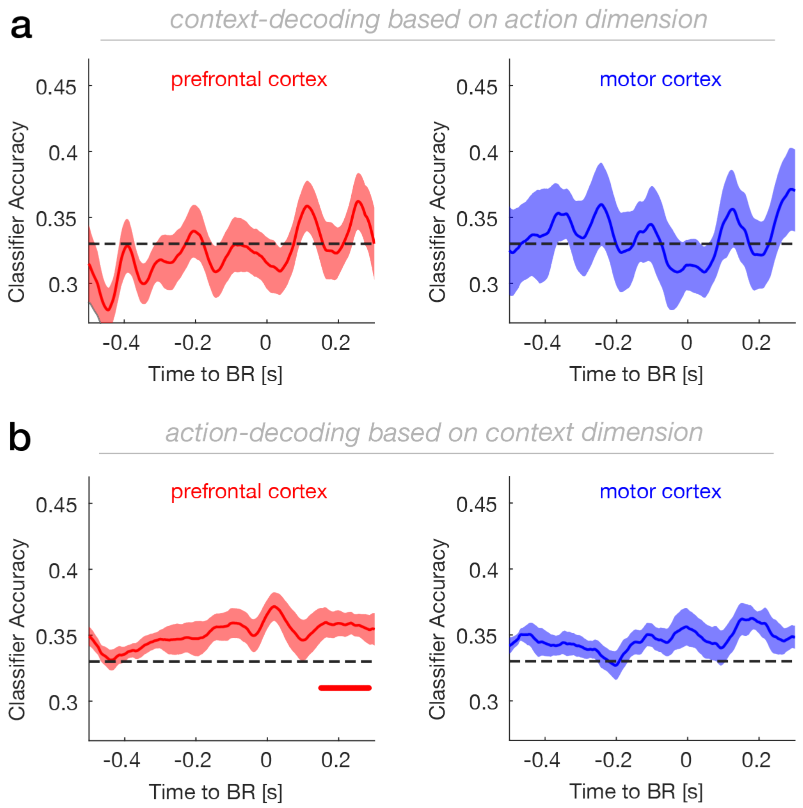

Supplementary Figure 10. Subspaces maximally discriminating context and action capture dissociable processes. a Context could not be successfully decoded in neither PFC (left; n = 14) nor motor cortex (right; n = 10) based on the dimension maximally discriminating reaction times (no cluster at $p < 0.05$). This suggests that the dimensions maximally dissociating context and action reflect dissociable processes. **b** Action-decoding based on the context-dimension was still possible in PFC ($t(13) = 201.28$, $p = 0.028$, Cohen's $d = 0.77$, n = 14; cluster test), but not in motor cortex ($t(9) = 121.5$, $p = 0.055$, n = 10). Note, however, that significant action-decoding was only possible after the action has already been executed, possibly reflecting action-outcome monitoring[3-6]. Source data are provided as Source Data file.

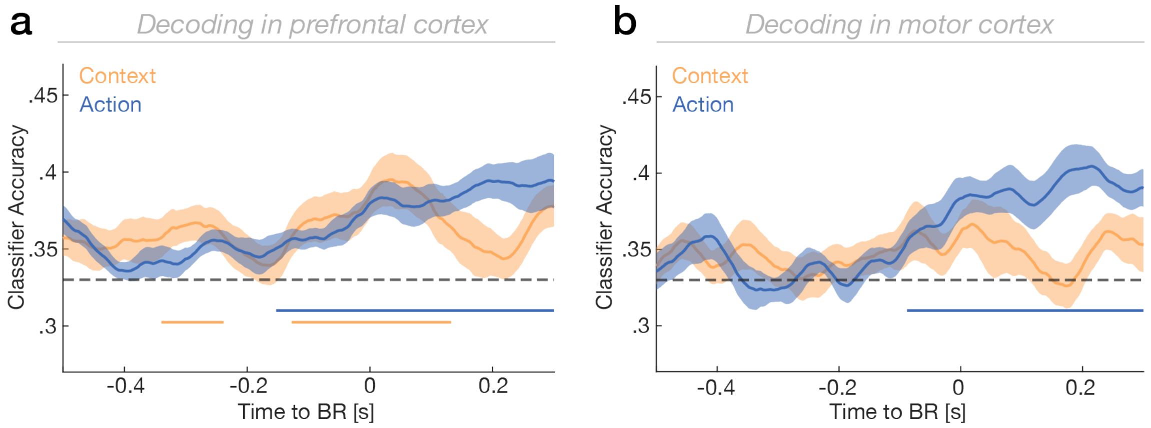

Supplementary Figure 11. Decoding analysis of context and action in high-dimensional space. a Grand average decoding accuracy (mean ± SEM) for context and action in PFC (n = 17). Classification was performed using all principal components cumulatively explaining more than 95% of the variance. Horizontal lines indicate the extent of significant temporal clusters (color coded for the respective feature; cluster test). **b** Grand average decoding accuracy (mean ± SEM) for context and action in motor cortex (n = 14). Only action, but not context, could be decoded in motor cortex. Same conventions as in **(a)**. Source data are provided as Source Data file.

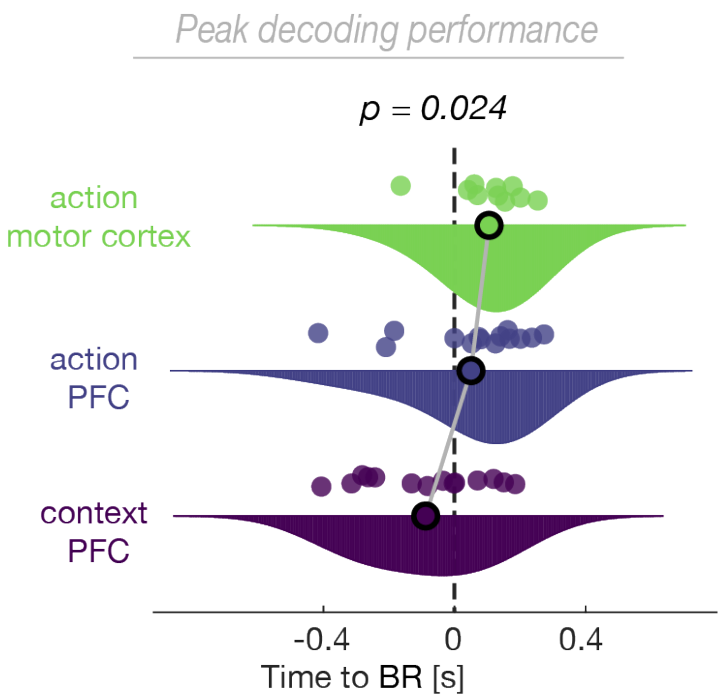

Supplementary Figure 12. Peak-decoding latency for context and action. Onset of peak-decoding performance demonstrating that initial context information in PFC serially unfolds into action encoding within the prefrontal-motor network (p = 0.024; Kruskal-Wallis; PFC context = -0.09 ± 0.19s.; PFC action = 0.05 ± 0.19s.; motor cortex action = 0.11 ± 0.12s; mean ± SD). Source data are provided as Source Data file.

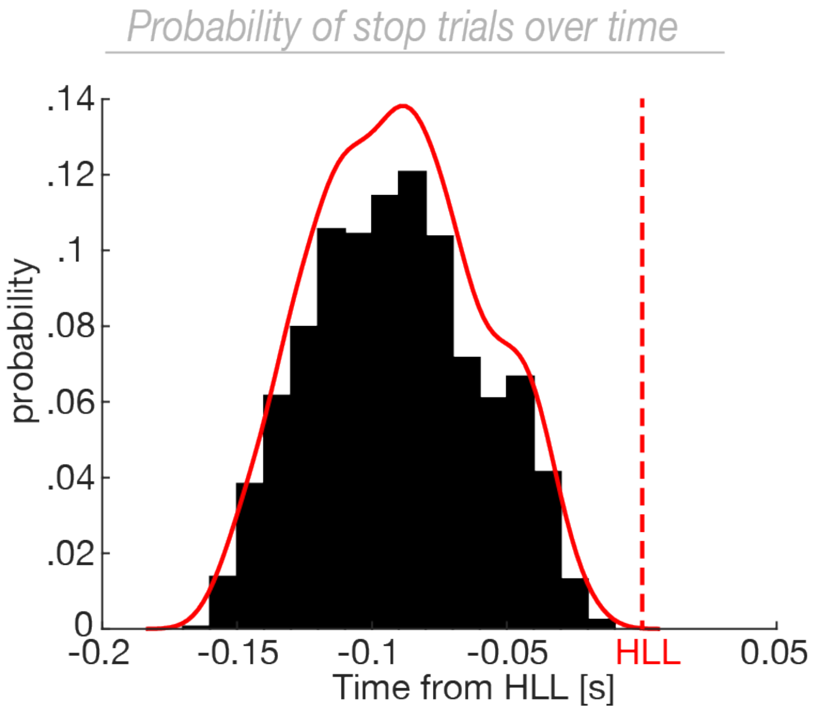

Supplementary Figure 13. Timing of stop trials in uncertain conditions. The probability of stop trials over time is depicted with respect to the predefined lower limit (HLL). The short time window between the probability of a target stopping and the HLL reinforced participants to integrate the predictive cue to guide their motor decisions. Source data are provided as Source Data file.

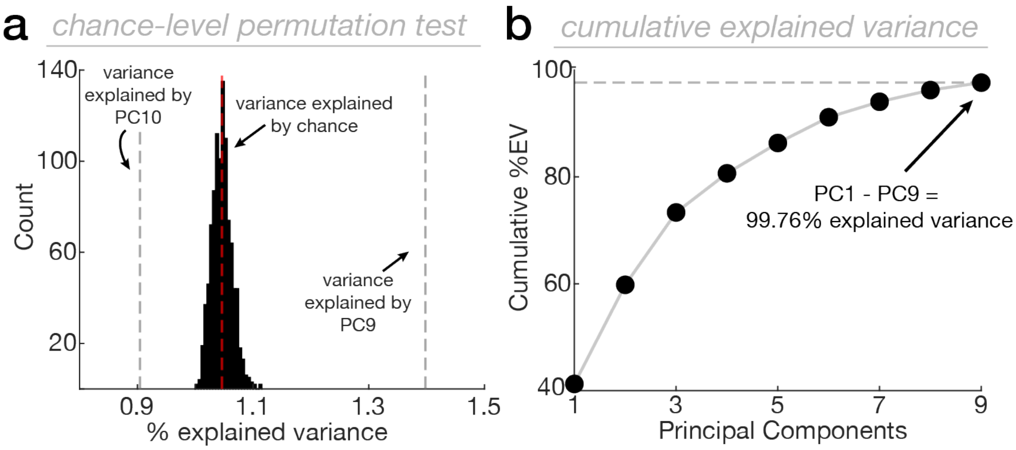

Supplementary Figure 14. Distinguishing significant from non-significant components. a Permutation distribution of explained variance by randomly shuffling the data. The red dashed line indicates the mean variance explained by chance. Principal components (PC) that explained more variance as expected by chance were considered to be significant. PC9 was the final component to be considered significant as it still explained more variance as to be expected by chance. **b** PC 1-9 cumulatively explained 99.76% of the variance.

Supplementary Table 1

PFC	*df*	*F*	*P*	R^2
RT ~ Peak Timing	1,2903	61.74	5.47×10^{-15}	0.021
RT ~ Peak Amplitude	1,2903	30.64	3.38×10^{-8}	0.01

Motor Cortex	*df*	*F*	*P*	R^2
RT ~ Peak Timing	1,2017	69.51	1.38×10^{-16}	0.033
RT ~ Peak Amplitude	1,2017	6.88	0.008	0.0029

Results for partial linear regression between HFA peak amplitude or peak timing and behavioral response time.

Supplementary Table 2

Modelled	*Parameter*	*Beta*	*T-statistic*	*df*	*p-value*	*Lower-95 CI*	*Upper-95 CI*	*Random effects (SD)*	*Model comparison (Likelihood ratio / p-value)*
HFA peak amplitude (simple model)	Intercept	7.416	17.921	75	0	6.592	8.240	1.29	0.87 / 0.34 (favoring simple model)
	ROI	0.179	1.469	75	0.146	-0.064	0.423		
	Prediction	0.012	3.522	75	**< 0.001**	0.005	0.019		
HFA peak timing (simple model)	Intercept	-0.103	-9.014	77	< 0.001	-0.126	-0.080	0.017	0.05 / 0.81 (favoring simple model)
	ROI	0.015	3.179	77	**0.002**	0.005	0.024		
	Prediction	0.0003	2.478	77	**0.015**	< 0.0001	0.0006		
HFA ramping dynamics (simple model)	Intercept	0.3	0.635	77	0.526	-0.639	1.240	0.934	0.37 / 0.54 (favoring simple model)
	ROI	0.029	0.158	77	0.874	-0.338	0.397		
	Prediction	0.012	2.149	77	**0.034**	0.0008	0.023		
Aperiodic slope (simple model)	Intercept	-2.886	-28.27	78	0	-3.089	-2.683	0.271	0.38 / 0.53 (favoring simple model)
	ROI	0.111	3.232	78	**0.001**	0.042	0.18		
	Prediction	0.001	0.984	78	0.328	-0.001	0.003		
Inter-peak-interval (simple model)	Intercept	8.232	162.88	77	0	8.131	8.332	0	0.54 / 0.46 (favoring simple model)
	ROI	-0.003	-0.159	77	0.873	-0.047	0.04		
	Prediction	-0.001	-2.561	77	**0.012**	-0.003	-0.0004		
PSI	Intercept	0.098	0.719	40	0.47	-0.177	-0.374	0.29	no other model used
	Prediction	0.005	2.221	40	**0.032**	0.0004	0.009		

Linear mixed-effect models supporting the results obtained from the ANOVAs (two-tailed).

References

1 Hipp, J. F., Hawellek, D. J., Corbetta, M., Siegel, M. & Engel, A. K. Large-scale cortical correlation structure of spontaneous oscillatory activity. *Nat Neurosci* **15**, 884-890, doi:10.1038/nn.3101 (2012).

2 Kaufman, M. T. *et al.* The Largest Response Component in the Motor Cortex Reflects Movement Timing but Not Movement Type. *eNeuro* **3**, doi:10.1523/ENEURO.0085-16.2016 (2016).

3 Gehring, W. J. & Knight, R. T. Prefrontal-cingulate interactions in action monitoring. *Nat Neurosci* **3**, 516-520, doi:10.1038/74899 (2000).

4 Luk, C. H. & Wallis, J. D. Dynamic encoding of responses and outcomes by neurons in medial prefrontal cortex. *J Neurosci* **29**, 7526-7539, doi:10.1523/JNEUROSCI.0386-09.2009 (2009).

5 Alexander, W. H. & Brown, J. W. Medial prefrontal cortex as an action-outcome predictor. *Nat Neurosci* **14**, 1338-1344, doi:10.1038/nn.2921 (2011).

6 Spellman, T., Svei, M., Kaminsky, J., Manzano-Nieves, G. & Liston, C. Prefrontal deep projection neurons enable cognitive flexibility via persistent feedback monitoring. *Cell* **184**, 2750-2766 e2717, doi:10.1016/j.cell.2021.03.047 (2021).
