# Supplementary material for: Ramping dynamics and theta oscillations reflect dissociable signatures during rule-guided human behavior
Source: Nat Commun. 2024 Jan 20;15:637. doi: 10.1038/s41467-023-44571-7 (PMC10799948; doi:10.1038/s41467-023-44571-7)
Supplement: Supplementary file 2 — Reporting Summary [file 41467_2023_44571_MOESM2_ESM.pdf]

nature portfolio

Corresponding author(s): Randolph Helfrich

Last updated by author(s): Oct 23, 2023

Reporting Summary

Nature Portfolio wishes to improve the reproducibility of the work that we publish. This form provides structure for consistency and transparency in reporting. For further information on Nature Portfolio policies, see our Editorial Policies and the Editorial Policy Checklist.

Statistics

For all statistical analyses, confirm that the following items are present in the figure legend, table legend, main text, or Methods section.

n/a	Confirmed	
☐	☒	The exact sample size (*n*) for each experimental group/condition, given as a discrete number and unit of measurement
☐	☒	A statement on whether measurements were taken from distinct samples or whether the same sample was measured repeatedly
☐	☒	The statistical test(s) used AND whether they are one- or two-sided<br>*Only common tests should be described solely by name; describe more complex techniques in the Methods section.*
☐	☒	A description of all covariates tested
☐	☒	A description of any assumptions or corrections, such as tests of normality and adjustment for multiple comparisons
☐	☒	A full description of the statistical parameters including central tendency (e.g. means) or other basic estimates (e.g. regression coefficient) AND variation (e.g. standard deviation) or associated estimates of uncertainty (e.g. confidence intervals)
☐	☒	For null hypothesis testing, the test statistic (e.g. *F*, *t*, *r*) with confidence intervals, effect sizes, degrees of freedom and *P* value noted<br>*Give P values as exact values whenever suitable.*
☒	☐	For Bayesian analysis, information on the choice of priors and Markov chain Monte Carlo settings
☒	☐	For hierarchical and complex designs, identification of the appropriate level for tests and full reporting of outcomes
☐	☒	Estimates of effect sizes (e.g. Cohen's *d*, Pearson's *r*), indicating how they were calculated

Our web collection on statistics for biologists contains articles on many of the points above.

Software and code

Policy information about availability of computer code

Data collection	Intracranial EEG data were acquired at the Oslo University Hospital at a sampling frequency of 512 Hz using the NicoletOne (Nicolet, Natus Neurology Inc., USA) or at a sampling frequency of 16 KHz using the ATLAS (Neuralynx) recording system.
Data analysis	The data was analyzed using Matlab 2021a (MathWorks Inc.), the toolbox Fieldtrip (accessed in September 2020; https://www.fieldtriptoolbox.org/) and custom code. Custom code used for this study will be made available at: https://github.com/JanWeber-neuro/Weber_iEEG_NatCommun.

For manuscripts utilizing custom algorithms or software that are central to the research but not yet described in published literature, software must be made available to editors and reviewers. We strongly encourage code deposition in a community repository (e.g. GitHub). See the Nature Portfolio guidelines for submitting code & software for further information.

Data

Policy information about availability of data

All manuscripts must include a data availability statement. This statement should provide the following information, where applicable:

- Accession codes, unique identifiers, or web links for publicly available datasets
- A description of any restrictions on data availability
- For clinical datasets or third party data, please ensure that the statement adheres to our policy

Source data are provided with this paper. The raw data are protected and are not openly available due to data privacy laws, though (subject to these privacy laws) the data are available upon reasonable request from Anne-Kristin Solbakk (a.k.solbakk@psykologi.uio.no) or Tor Endestad (tendesta@uio.no). A reporting summary

for this article is available as a Supplementary Information file. The atlas used for electrode classification (ROI_MNI_V4.nii) can be accessed via the Fieldtrip Toolbox (https://www.fieldtriptoolbox.org/).

Research involving human participants, their data, or biological material

Policy information about studies with human participants or human data. See also policy information about sex, gender (identity/presentation), and sexual orientation and race, ethnicity and racism.

Reporting on sex and gender	19 pharmaco-resistant epilepsy patients (33.73 years ± 12.52, mean ± SD; 7 females)
Reporting on race, ethnicity, or other socially relevant groupings	N/A
Population characteristics	Human epilepsy patients who were implanted with intracranial depth electrodes for seizure onset localization.
Recruitment	Patient recruitment involved asking those who were implanted with depth electrodes in motor cortex and/or prefrontal cortex (regions of interest in the present study). Patients were directly recruited at the Department of Neurosurgery, Oslo University Hospital.
Ethics oversight	All procedures were approved by the Regional Committees for Medical and Health Research Ethics, Region North Norway (#2015/175) and the Data Protection Officer at the Oslo University Hospital as well as the University Medical Center Tuebingen (049/2020BO2) and conducted in accordance with the Declaration of Helsinki.

Note that full information on the approval of the study protocol must also be provided in the manuscript.

Field-specific reporting

Please select the one below that is the best fit for your research. If you are not sure, read the appropriate sections before making your selection.

- [x] Life sciences
- [ ] Behavioural & social sciences
- [ ] Ecological, evolutionary & environmental sciences

For a reference copy of the document with all sections, see nature.com/documents/nr-reporting-summary-flat.pdf

Life sciences study design

All studies must disclose on these points even when the disclosure is negative.

Sample size	No sample size calculation was performed. Participants were recruited continuously from the Department of Neurosurgery, Oslo University Hospital depending on the electrode coverage. We included 18 participants which exceeds the number of participants reported in comparable studies (e.g. Voytek et al. 2015 Nat. Neurosci.; Kaminski et al. 2017 Nat. Neurosci.; ter Wal et al. 2022 Nat. Commun.).
Data exclusions	Data from one patient were excluded from neural analyses because a low-pass filter was applied at 50 Hz during data export from the clinical system. We further considered participant data where z-scores exceeded 3rd standard deviation as outliers.
Replication	Behavioral and within-subject electrophysiological effects were replicated across different participants. No replication was performed for group-level analyses. Low dimensional representations of overt behavior in motor cortex were previously reported in non-human primate models and were replicated here in human motor cortex.
Randomization	All participants performed all three experimental conditions, which were randomly presented. Hence, we employed a repeated-measures design and participants were not assigned to different experimental conditions, hence, randomization at group level was not applicable.
Blinding	Participants were unaware of the main hypotheses of the experiment. Participants were not allocated to groups during data collection and analysis (within-subject design). Thus, no blinding of experimenters to group allocation was necessary.

Reporting for specific materials, systems and methods

We require information from authors about some types of materials, experimental systems and methods used in many studies. Here, indicate whether each material, system or method listed is relevant to your study. If you are not sure if a list item applies to your research, read the appropriate section before selecting a response.

Materials & experimental systems

n/a	Involved in the study
☒	☐ Antibodies
☒	☐ Eukaryotic cell lines
☒	☐ Palaeontology and archaeology
☒	☐ Animals and other organisms
☒	☐ Clinical data
☒	☐ Dual use research of concern
☒	☐ Plants

Methods

n/a	Involved in the study
☒	☐ ChIP-seq
☒	☐ Flow cytometry
☒	☐ MRI-based neuroimaging

Plants

Seed stocks	N/A
Novel plant genotypes	N/A
Authentication	N/A
